# Genome-wide association analysis of resistance to bacterial cold-water disease in an important rainbow trout aquaculture breeding population

**DOI:** 10.3389/fgene.2025.1582138

**Published:** 2025-09-18

**Authors:** Bruna Santana, Yniv Palti, Guangtu Gao, Vibha Tripathi, Kyle E. Martin, Breno O. Fragomeni

**Affiliations:** ^1^ Animal Genomics Laboratory, Department of Animal Science, University of Connecticut, Storrs, CT, United States; ^2^ National Center for Cool and Cold Water Aquaculture, United States Department of Agriculture, Agricultural Research Service, Leetown, WV, United States; ^3^ Hendrix Genetics, Sumner, WA, United States

**Keywords:** rainbow trout, disease resistance, genomic predictions, oligogenic trait, quantitative trait loci, prediction accuracy

## Abstract

Bacterial cold-water disease (BCWD) outbreaks in salmonid aquaculture have resulted in significant losses in commercial populations. Currently, there is no commercially available vaccine for the disease caused by *Flavobacterium psychrophilum*. BCWD resistance in rainbow trout exhibits moderate heritability and has been the focus of selection efforts. The understanding of key genomic regions associated with BCWD resistance has advanced since the integration of genomic information into genetic evaluations, proving successful in enhancing BCWD resistance in some commercial lines. Here, we report the results of a genome-wide association study for BCWD resistance in an important commercial rainbow trout line to further our understanding of the genetic architecture of the trait and infer a selective breeding strategy for this line. Different scenarios were tested, including the use of all single-nucleotide polymorphisms (SNPs) passing quality control, removal of SNPs with major effect, elimination of consistent “major SNPs” in subgroups of the population, and exclusion of SNPs within haplotypes with major effect. Prediction accuracy was evaluated with different SNP weighting strategies, utilizing cross-validation groups formed either randomly or based on principal components and cluster analyses of genotypic data. Comparative analysis of cross-validation methods suggested that partitioning of the dataset using K-means clustering reduced overfitting. The incorporation of SNP weighting further confirmed the oligogenic nature of the trait under investigation. Prediction accuracy with pedigree-based best linear unbiased prediction (PBLUP) was 0.27 and increased to 0.36 with genomic information. The accuracy obtained with a single largest effect haplotype was 0.23. Moreover, a decrease in accuracy was observed upon excluding major SNPs and haplotypes, providing supplementary evidence of their importance on phenotypes. The two largest association peaks on OmyA31/Omy25 and Omy8 were consistent with previous reports.

## 1 Introduction

Bacterial cold-water disease (BCWD) outbreaks in salmonid aquaculture lead to morbidity (Madsen and Dalsgaard, 1999; Nilsen et al., 2011) and high mortality rates across populations (Dalsgaard and Madsen, 2000; Nematollahi et al., 2003; Loch and Faisal, 2015). The disease is caused by a treatable bacterium, *Flavobacterium psychrophilum*. However, the use of antibiotics may increase production costs, environmental build-up, and promote the selection of pathogen strains that may be resistant to antibiotics. Vaccination is another form of addressing the issue by improving the protection of immunocompetent fish (Hoare et al., 2017; 2019). However, at present, there is no commercially available vaccine for BCWD (Vallejo et al., 2022). Genomic selection has been proved successful in substantially improving BCWD resistance in rainbow trout, and for this reason, it has been adopted for commercial implementation (Vallejo R. et al., 2017; 2018). In recent years, following the development of improved genome resources for rainbow trout (Palti et al., 2015b; Gao et al., 2021), many more reports have been published from a variety of labs reporting on the use of genome-wide association studies and the use of genomic-enabled predictions for selection in resistance to other pathogens and for other important aquaculture production traits (Ali et al., 2019; Reis Neto et al., 2019; Silva et al., 2019; Yoshida et al., 2018; Ahmed et al., 2022; Calboli et al., 2022; Fraslin et al., 2022; 2023; Garcia et al., 2023; Kudinov et al., 2024; Palti et al., 2024).

Typical statistical approaches in genomic selection often assume every locus to have influence over complex traits (Meuwissen et al., 2001; VanRaden, 2008; Aguilar et al., 2010). This assumption is suitable for polygenic traits (Santana et al., 2023) and large data sets (Lourenco et al., 2017). However, there are scenarios when using variable selection methods (Mehrban et al., 2017) or an SNP weighting approach (Vallejo et al., 2019) will lead to higher accuracies. By assigning weights to single-nucleotide polymorphisms (SNPs) during the construction of the genomic relationship matrix, it is possible to ease the assumption that all markers have a uniform contribution to the phenotypic expression (VanRaden, 2008). Alternative assumptions in terms of the distribution of the markers’ effects were beneficial for accounting for particular genetic architectures, especially for oligogenic traits (Wang et al., 2012; Zhang et al., 2016; Fragomeni et al., 2017, 2019; Lourenco et al., 2017).

Genomic regions identified as significant for BCWD have been consistent across studies in rainbow trout. Two major quantitative trait loci (QTL) on chromosomes Omy08 and Omy25 were shown to explain more than 10% of the additive genetic variance for BCWD resistance (Liu et al., 2018; Vallejo R. L. et al., 2017). Furthermore, a QTL on Omy25 was validated in an independent population, and individuals carrying the favorable allele exhibited a significantly higher survival rate (Mathiessen et al., 2023). Important associations identified in genome-wide association studies (GWAS) are not expected to change drastically over several generations in a population until recombination can start to break down the association between genetic markers and the causative QTL variant. Additionally, exceptions might occur when divergent individuals are introduced into the population (Uffelmann et al., 2021), in polygenic traits (Fragomeni et al., 2014), or due to allele fixation (Muir et al., 2008).

Troutlodge, Inc., the largest distributor of rainbow trout eggs in the United States and worldwide, maintains a year-round production of eggs using four distinct broodstock lines, with peak spawning in February, May, August, and November. Phylogenetic analyses based on genotypes from 96 Fluidigm SNP assays indicated that all four lines are genetically distinct (Liu et al., 2017). Previously, we identified two significant QTL for BCWD resistance on chromosomes Omy08 and Omy25p_OmyA31 in the May spawning line (Vallejo R. L. et al., 2017), which have been shown to be effective in a marker-assisted selection (MAS) breeding strategy in that line (Liu et al., 2018; 2022).

Genome-wide association studies (GWAS) may be adopted to elucidate a trait’s genetic architecture (Goddard et al., 2016). As a consequence, they may provide better insights about assumptions of SNP effect’s distribution, and additionally, be used to improve SNP array design (Moghaddar et al., 2022), target genomic regions for gene editing (Lee and Fidock, 2014), inform drug development programs (Uffelmann et al., 2021), and for MAS (Liu et al., 2018).

In the present study, we conducted genomic analyses in the Troutlodge February spawning nucleus breeding population, which represents a distinct genetic line that is equally important to the U.S. aquaculture industry as the previously studied Troutlodge May line. Hence, the objective of this study was to perform GWAS for BCWD resistance in this distinct and commercially important rainbow trout line while adopting distinct approaches to compute SNP effects, methods to validate major SNPs, and identify haplotypes associated with the trait. Additionally, this study aimed to predict accuracy when different SNP weighting strategies were adopted to elucidate the most effective selective breeding strategy for this commercial rainbow trout line.

## 2 Materials and methods

### 2.1 Ethics statement

This study used rainbow trout fin clips collected after controlled infection with *Flavobacterium psychrophilum (Fp)* at the Center for Aquaculture Technology (CAT) research facility (Souris, PE, Canada) as part of the Troutlodge, Inc., selective breeding program. As farm animals used in a commercial breeding program, these fish are exempt from regulation under the U.S. Animal Welfare Act and, therefore, not subject to oversight by an Institutional Animal Care and Use Committee or other such ethics committee. This exemption is defined in U.S. Code title 7, chapter 54, section 2132g. However, experimentation and handling were conducted in accordance with U.S. government principles for the use and care of vertebrate animals used in testing, research, and training, which include provisions to minimize animal suffering. Specific measures for amelioration of animal suffering during the fish pathogen testing included minimization of handling and maintenance of optimal water temperature and oxygen saturation; additionally, the fish were fed a standard fish meal diet to satiation daily. Fish near death from severe symptoms of infection during the observation period were removed and terminated (by immersion in a lethal dose of MS-222) before the collection of fin tissue to minimize suffering. After the 24-day observation period, surviving fish were terminated by immersion in a lethal dose of 500 mg/L of MS-222 for 30 min before sampling and disposal.

### 2.2 Fish rearing and disease challenge

Equal volumes of ∼30 eyed eggs per full-sib (FS) family from 148 families were pooled randomly at the Troutlodge hatchery facility in Sumner, Washington, United States, and shipped overnight to the research facility of the Center for Aquaculture Technologies in Canada (Souris, PE). Throughout all phases of the study, water was maintained with 90%–130% oxygen saturation. Flow through freshwater maintained at 8 °C with a daily fluctuation of ±1 °C was used during the egg incubation and hatching phases. Heath stacks remained covered throughout incubation, and dead eggs were removed daily. Once yolk sacs were absorbed, fry were transferred to larger holding units and were held under a 24-h light photoperiod in recirculating 12 °C ± 1 °C freshwater, at flow rates set to allow for adequate flushing/waste removal and maintenance of environmental parameters. The fish were fed to satiation twice daily. At 80 days post hatching, equal numbers of fry were divided to three replicate tanks for the BCWD challenge and 24-day survival trial. Average body weight on the first day of the disease challenge was 1.52 g with a standard deviation of ±0.44 g. The disease challenge protocol developed by the USDA-ARS group from Leetown, WV, United States (Silverstein et al., 2009; Leeds et al., 2010), was followed in this study. Fish within a respective holding tank were grouped into cohorts of N = 50 and anesthetized via immersion prior to being intraperitoneally (IP) injected with 0.1 mL of PBS with the *F. psychrophilum* bacterial strain CSF-295-93 (4.7 × 10^6^ CFU/fish). This is a pathogenic strain of the bacteria that has been shown to cause consistent mortality in the lab challenge model, with a strong correlation with survival performance after outbreaks in the field. The strain was isolated by Dr. Scott LaPatra in 1993 from a farm outbreak in Idaho, United States. It was used to derive a live attenuated vaccine and has been characterized biologically and immunologically (Lafrentz et al., 2007; Lafrentz et al., 2009; Lafrentz et al., 2011; Sudheesh et al., 2007). The genome of this strain was sequenced and characterized by Wiens et al. (2014). The bacterial strain isolate, with specific storage conditions and culture know-how information, can be obtained upon request and appropriate arrangements for research material transfer from the corresponding author. The injection process took approximately 2 h per holding tank, or 6–7 h in total. Once injected, fish were placed in their respective holding tank for recovery and observation. The sham control group included two random cohorts, each composed of 50 fish from the pool of 148 families that were injected with PBS and kept at similar density and water conditions to the larger three replicate tanks with the bacteria-injected fish throughout the 24-day survival trial. No mortalities were observed in the sham control group. Fish welfare checks were performed twice daily during the growth phase from hatching to day 80 post hatching and following the disease challenge. Dead and moribund fish were removed, recorded, and fin clip samples were taken from the removed fish daily during the 24-day survival trial following the disease challenge. Fin clips from the sampled fish were stored individually in 95% ethanol at room temperature for up to 2 months until DNA extractions.

The dose used in the injections was identified empirically to accomplish an approximately 50% survival rate per tank, which is the typical goal of disease resistance studies that aim to maximize phenotypic diversity in order to exploit the genetic diversity in the population. Previous work by the USDA group has shown that for Fp infection to induce BCWD in rainbow trout, the IP injection model provided the most consistent results across challenges, populations and over time and that significant response to selection using the lab IP model corresponded with significant improvement in survival after disease outbreaks in the field (Silverstein et al., 2009; Leeds et al., 2010; Wiens et al., 2013a; Wiens et al., 2018). It has also been a very successful model for detecting QTL and for developing genomic selection predictions with very good accuracy (Vallejo et al., 2022; Liu et al., 2022; Vallejo et al., 2018; Vallejo R. et al., 2017) and has been used to analyze gene expression responses to infection in the host (Marancik et al., 2015; Wiens et al., 2023).

### 2.3 Data

Phenotypic data for days to death (DAYS) were collected from 3,784 rainbow trout fish. The number of fish with survival phenotype data collected from tanks C1, C2, and C3 were 1,279, 1,210, and 1,295, respectively. DAYS is a discrete variable representing the number of post-challenge days survived in a period of 24 days. All survivors at the end of the challenge were assigned the phenotype of 24 days. A total of 3,784 DAYS records were collected, with an average value of 16.32 and a standard deviation (SD) of 7.68. The average (SD) DAYS per tank were 16.86 (7.59), 16.29 (7.75), and 15.84 (7.69). There were no significant differences between tanks 1 and 2 (p > 0.05), and tank 3 was significantly different from the other two tanks (p < 0.05) with a difference of 1.02 DAYS according to two-sample t-tests. Therefore, comparisons between outcomes from models with and without the tank effect were conducted and will be included in the next section. The fish with phenotypes were assigned to 141 full-sib families using a low-density SNP array following previously described methods (Liu et al., 2016; Vallejo et al., 2022). The median number of fish per family was 28, with an average of 26.8, a minimum of 1, and a maximum of 38. The overall pedigree file contained 6,658 individuals spanning over 12 generations.

A total of 2,121 fish from 141 families were genotyped by a commercial genotyping service provider (Center for Aquaculture Technologies, San Diego, CA) using the rainbow trout 57K Axiom Genotyping SNP Array 384-well format (Thermo Fisher Scientific catalog number 550571) (Palti et al., 2015a). Genotype calls were done by the authors using the Affymetrix Array Power Tools kit for the rainbow trout Axiom array, followed by bioinformatics processing of the data for quality control (QC) and formatting for the BLUPF90 analysis using standard methods as previously described (Palti et al. 2015a; Vallejo R. et al., 2017). Of the genotyped animals, 1,897 had phenotypic records with a mean of 16.28 DAYS and a standard deviation of 8.28. A total of 1,231, 224, and 442 individuals were genotyped from tanks C1, C2, and C3, respectively. All the animals with phenotypes from tank C1 were genotyped. The animals genotyped from tanks C2 and C3 were selected from families of greater importance for the breeding program due to high genetic merit predictions for other production traits. The remaining 224 genotyped animals had no phenotype records and were the ancestors of the individuals in the challenge.

A total of 36,676 SNP markers passed QC filtering. The dataset of filtered markers provided good coverage for all 32 chromosomes in the reference rainbow trout genome. During QC, samples and markers with a call rate of less than 0.90 or with 10% or more Mendelian conflicts were removed. Additionally, markers with minor allele frequency (MAF) less than 0.05 were also removed. Finally, markers from unplaced contigs and the mitochondria were excluded from the analysis.

### 2.4 Model and analyses

Pedigree and genomic breeding values (estimated breeding values (EBV) and genomic estimated breeding values (GEBV), respectively) for DAYS were calculated from the following linear model (Equation 1):

y=Xb+Za+e, (1)

where y is the vector of phenotypes (DAYS); b is the vector of fixed effects, which includes the overall mean; a is a vector of random individual additive genetic effects; e represents the random residuals; and X and Z are the incidence matrices for vectors b and a , respectively.

In a second model, potential predominant haplotype(s), which would be closely aligned with a specified major SNP identified in GWAS, were incorporated as an additional fixed effect as a covariate. Details of the haplotype identification process are provided in Section 2.6. Model 2 (Equation 2) had a fixed effect that distinguishes groups based on the presence of none, one, or two haplotype copies of the highly associated haplotype(s), and **W** is the incidence matrix for the hp vector:

y=Xb+Whp+Za+e. (2)

In Model 2, the variance explained by the detected haplotype was computed to account for changes in the additive genetic variance due to the inclusion of the haplotype effects: ${hp}_{i}^{2}2p_{i}q_{i}$ , where $hp_{i}$ is the estimated fixed effect of the *ith* haplotype.

The assumption of the variances of the random effects was

$$y\;∼\text{ MVN}=\left[\mu ,\text{var}\right],$$

where μ = $\left[\begin{array}{c} 0\\ 0 \end{array}\right]$ , $\text{var}\left[\begin{array}{c} a\\ e \end{array}\right]=\left[\begin{array}{cc} A\sigma_{a}^{2} & 0\\ 0 & I\sigma_{e}^{2} \end{array}\right],$ $\sigma_{a}^{2}$ is the additive genetic variance, $\sigma_{e}^{2}$ is the residual variance, **A** is the numerator relationship matrix (NRM), and **I** is the identity matrix. This approach, where only pedigree information was used to model the covariance between individuals, will be referred to throughout this document as PBLUP. The assumptions are modified to include genomic information under a single-step GBLUP approach (ssGBLUP), which is the method of choice when not all animals are genotyped (Aguilar et al., 2010; Christensen and Lund, 2010). The ssGBLUP approach reduces biases in populations under selection by properly weighting information obtained from pedigree and phenotypes (Vitezica et al., 2011; Legarra et al., 2014). This method uses the same models as traditional pedigree-based evaluations, and the genomic information is incorporated by replacing the NRM with the **H** matrix, which includes full pedigree and genomic relationships (Legarra et al., 2009). The matrix **H**, as defined by Legarra et al. (2009), is its inverse, which can be obtained as in Aguilar et al. (2010):

$$H^{-1}=A^{-1}-\left[\begin{array}{cc} 0 & 0\\ 0 & G^{-1}-A_{22}^{-1} \end{array}\right],$$

where $A^{-1}$ is the inverse of the numerator relationship matrix, $A_{22}^{-1}$ is the inverse of the numerator relationship matrix only for genotyped animals, and $G^{-1}$ is the inverse of the genomic relationship matrix, which was calculated as (VanRaden, 2008)

$$G=\frac{MM^{\prime }}{\sum_{j=1}^{m}2p_{j}q_{j}},$$

where **M** is a matrix of gene content, centered on the allele frequencies that are obtained from the entire genotyped population being evaluated, $p_{j}$ is the allele frequency of the major allele of the *j*th SNP, and $q_{j}=\left(1-p_{j}\right)$ .

The variance components analyses were performed with PBLUP and ssGBLUP under an AIREML approach for DAYS with Model 1. Breeding values were calculated by solving the system of equations using the variance components calculated in the previous step. All computations were performed using BLUPF90+ software (Misztal et al., 2015).

An additional model was tested, similar to Model 1, but without the fixed effect of the tank from which the fish were reared as a fixed effect with values ranging from 1 to 3. This model utilized complete phenotypic records under ssGBLUP. The GEBVs calculated under this model scenario correlated at 0.99 with those from Model 1. Therefore, only results from Model 1 without the tank effect are reported because there may be a risk of a confounding effect between genetics and tank.

### 2.5 Cross validation for predictive ability

Predictive ability was defined as the correlation between predicted and observed phenotype (Legarra et al., 2008) and was estimated using cross validation. The observed phenotypes were corrected by subtracting the fixed effects calculated in a preliminary run with complete data (Lourenco et al., 2015). The estimated breeding values (EBV) and genomic estimated breeding values (GEBV) were estimators of the phenotypes.

In an initial approach, genotyped animals were randomly assigned to five groups of equal size. In each cross-validation round, four of the groups were included in a training set and the one remaining group was used for validation. This step was repeated five times, so all genotyped fish would be included in the validation group once. The validation group’s phenotypes were masked and not used in the (g)EBVs computation. Full pedigree and genomic data were used for all animals. Fish without genotypes had their phenotypes included in the training groups. Predictive ability was the average correlation between corrected phenotypes and (g)EBV for the validation fish across the five random groups. Additionally, the regression coefficient (b1) of the corrected phenotype on the (g)EBVs was used to verify inflation (b1 > 1) or deflation (b1 < 1) of predictions.

In a second scenario, fish with identified genotypes were assigned to groups according to their genomic relationships based on principal components and K-means clustering of the genotypic data (Saatchi et al., 2011). K-means clustering is an algorithm used to group similar data points into clusters. In this study, it was used to generate groups of samples for cross validation compared to random grouping. Individuals exhibiting greater genetic similarity were grouped together. The training and validation procedures were similar to the previous approach, where four groups were in the training group and one was in the validation set each time. Similarly, ungenotyped fish with phenotypic information were included in the training groups. The numbers of records or genotypes for the cross-validation groups are presented in Table 1.

**TABLE 1:** Numbers of fish per cross-validation group for two scenarios. Group 6 is composed of the non-genotyped samples, which were included in the training set in all cases.

Scenario	1	2	3	4	5	6
K-means	316	143	549	677	437	1,886
Random	425	425	424	424	424	1,886

### 2.6 Haplotype identification and model inclusion

Haplotypes were identified with findhap.f90 version 3 (VanRaden et al., 2013). The program initially processes the haplotypes of close ancestors from the oldest to the youngest individuals. Genotypes are phased into haplotypes based on either ancestor haplotypes or the most common non-conflicting haplotype (VanRaden et al., 2015). Haplotypes of varying lengths were utilized as priors and to identify the ideal parameters (results not shown). The haplotypes were identified by checking segments between 20 and 2000 SNPs, and the maximum number of haplotypes allowed was 5000. The rate of miscalled genotypes allowed when matching haplotypes was 0.004. The haplotyping process was conducted over four iterations. The values used were based on preliminary runs and on the software-recommended values. The following options were used in findhap.f90: *iters* = 4, *maxlen* = 2000, *minlen* = 20, *step*s = 3, *maxhap* = 5000, and *errate* = 0.004.

Additionally, a simple linear model that included the intercept and the main haplotype effect was tested. This model differs from Model 2 as it does not include the random additive genetic effect. Moreover, in this model, the haplotype effect was tested as both a covariate and a categorical effect. Finally, the results of this linear model were summed to the PBLUP breeding values to mimic a marker-assisted selection with a single marker.

### 2.7 Genome-wide association analysis

The current rainbow trout reference genome (Gao et al., 2021) was used for the GWAS. The SNP effects were calculated based on the ssGBLUP solutions using a back-solving process derived by Wang et al. (2012), based on formulas provided by VanRaden (2008) and Strandén and Garrick (2009):

$$\hat{u}=\lambda DM^{\prime }G^{-1}\hat{a,}$$

where $\hat{u}$ is the vector of estimated SNP effects, λ is the ratio of SNP to genetic variance, **D** is a matrix of weights, and $\hat{a}$ is a vector of GEBV. Once SNP effects are calculated, this procedure can be iterated by assigning weights to SNPs to calculate a weighted **G** matrix, which can be obtained as

$$G_{w}=\frac{\text{MDM}}{\sum_{j=1}^{m}2p_{j}q_{j}}.$$

Then, $G_{w}$ was used to recalculate GEBV until convergence was achieved. The convergence criterion was met when the changes in GEBVs from the current to the previous iteration were less than 10^−4^. The diagonal elements of **D** were obtained by squaring $\hat{u}$ to $\hat{u^{2}}$ (Fragomeni et al., 2017). Finally, matrix **D** was normalized so that tr(**D**) = tr(**I**). This weighting approach will be referred to as quadratic weighting. Alternatively, weights were calculated using the NonlinearA approach proposed by VanRaden (2008). This approach considers a heavy-tailed distribution for SNP variance in an iterative method and avoids extreme shrinkage of SNP effects by limiting weights. The weights of SNP effects were calculated as

$$d_{i}={\text{CT}}^{\frac{\left|\hat{u_{i}}\right|}{sd\left(\hat{u}\right)}-2},$$

where $\left|\hat{u_{i}}\right|$ is the absolute estimated SNP effect for marker *i* and $sd\left(\hat{u}\right)$ is the standard deviation of the vector of estimated SNP effects. The values used as the constant were 1.125, 1.175, and 2. To avoid extreme weights, the maximum values of $\frac{\left|\hat{u_{i}}\right|}{sd\left(\hat{u}\right)}$ were limited to 10 and 50.

GWAS was performed using Model 1 and Model 2. The percentages of variance explained by windows of 10 adjacent SNPs were used for the Manhattan plots. They were calculated by summing the SNP individual variances and then dividing this value by the total additive genetic variance using software POSTGSF90 (Aguilar et al., 2014). Additionally, accuracy was calculated with alternative SNP arrays based on GWAS results: 1) All SNPs that passed quality control “Total SNP”; 2) removal of SNPs explaining more than 0.5% of the additive genetic variance; this scenario is referred to as “Minus Major SNP”; 3) removal of SNPs that explained more than 2% of the additive genetic variance and were consistent in all K-means cross-validation groups; this scenario is referred to as “Minus Common SNP”; 4) removal of SNPs within major haplotypes; this scenario is referred to as “Minus Haplotype.” The thresholds used for the SNP deletion were determined based on visual inspection of Manhattan plots. Normalized relationship matrices were obtained with average diagonal coefficients set to 1 for scenarios with additional exclusion of SNPs after quality control.

## 3 Results

### 3.1 BCWD survival phenotypes

**FIGURE 1:**
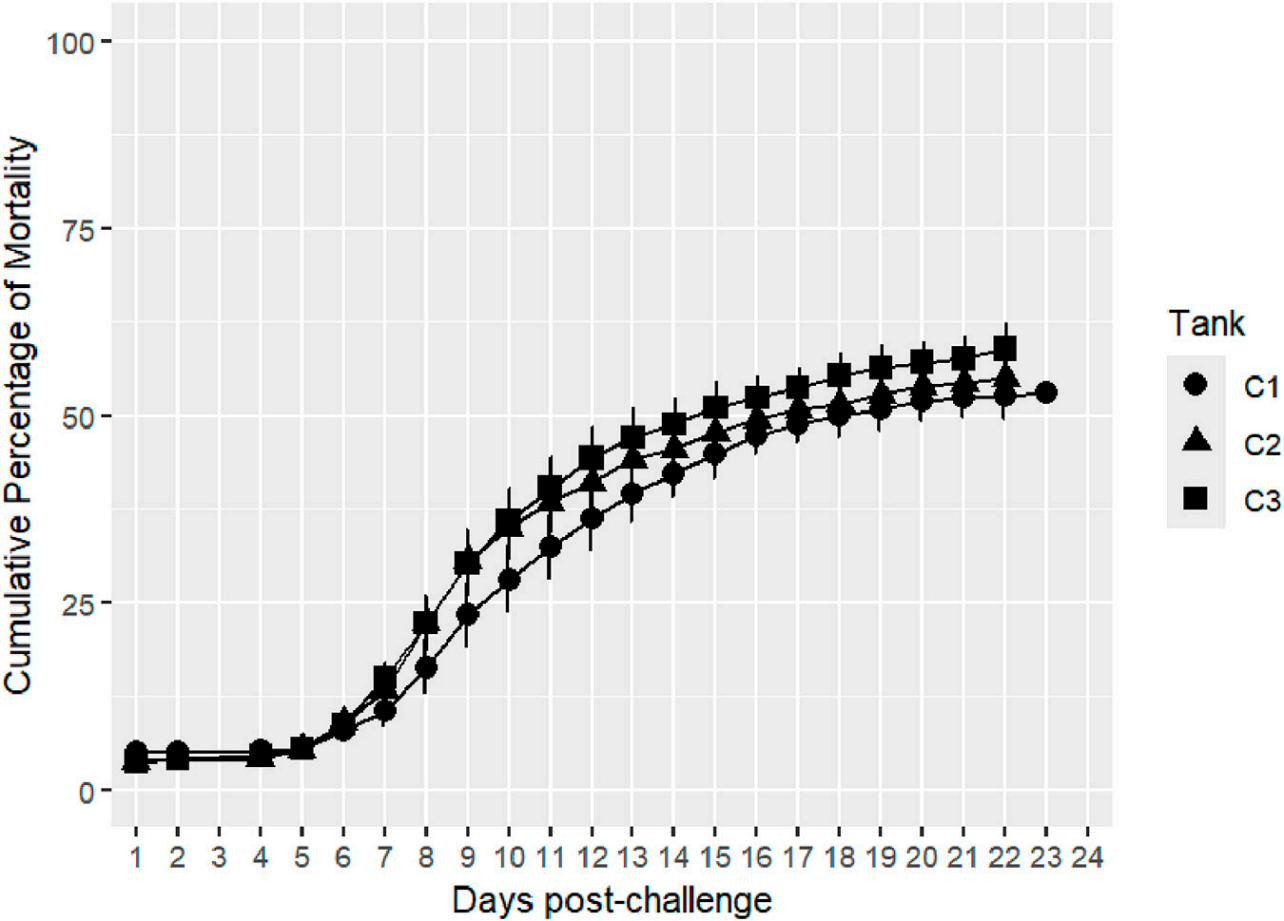
Distribution of daily mortality post-challenge in the commercial rainbow trout line. The bars represent the standard deviation (SD) between tanks.

**FIGURE 2:**
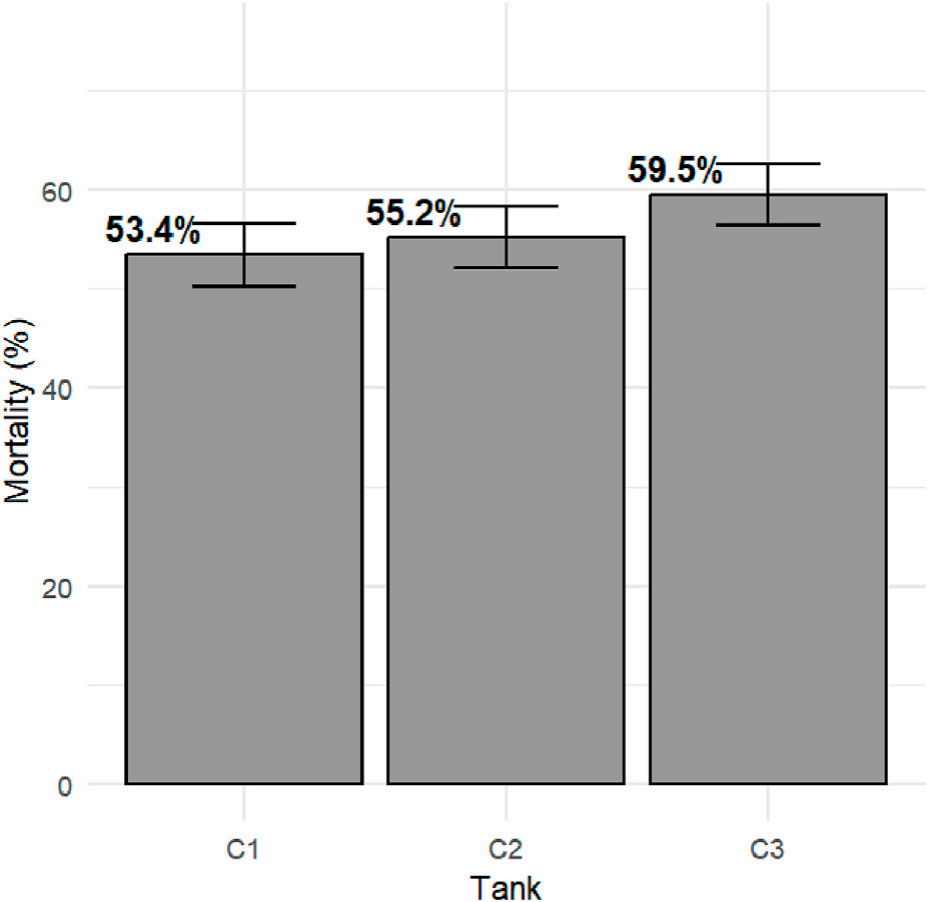
Total mortality post-challenge in the commercial rainbow trout line. The bars represent standard error (SE) between tanks.

Survival records were obtained from a total of 3,784 fish across three replicate tanks with slightly fewer than 1,300 fish per tank. Mortality with clinical signs of bacterial cold-water disease was first observed on day 5 of the challenge. The mortality kinetics were nearly identical across the three tanks, with peak mortality days between days 6 and 14 of the post-challenge survival trial. Mortality rate plateaued from day 16, reaching a cumulative mortality rate of ∼50%, and the last mortality was recorded on day 23 (Figure 1). Fish that survived to day 24 of the post-challenge survival trial were recorded as alive at the end of the trial. Average mortality rate per replicate tank was ∼55% with higher mortality observed in tank 3 (Figure 2). Survival days data were also recorded, with an average value of 16.32 days and an SD of 7.68. Fish that were alive at the end of the trial were assigned a survival days value of 24. The averages (SD) per tank were 16.86 (7.59), 16.29 (7.75), and 15.84 (7.69). There were no significant differences in survival days between tanks 1 and 2 (p > 0.05), and tank 3 was significantly different from the other two tanks (p < 0.05).

### 3.2 Variance components

Estimates of heritability are presented in Table 2. The heritability (SE) with PBLUP was 0.39 (0.05), and for ssGBLUP, the values were 0.30 (0.03) and 0.15 (0.04), using Model 1 and Model 2, respectively. The incorporation of haplotype copies as a fixed effect and the elimination of a major haplotype from the SNP panel caused the additive genetic variance of the trait to decline. The variance of the haplotype was 3.38, which would result in a heritability of 0.19 when that variance is incorporated into the genetic variance.

**TABLE 2:** Genetic parameter estimates for BCWD survival status in the rainbow trout February spawning commercial line of Troutlodge from the 2020 year class. 2020 with different statistical approaches, including the genetic additive variance component (σ_a_), residual component (σ_e_), haplotype effect (Hap^2^2pq), heritability (h^2^), and standard error (se).

Model	σ_a_ (se)	σ_e_ (se)	Hap^2^2pq	h^2^ (se)
PBLUP	24.38 (3.72)	37.58 (2.23)	-	0.39 (0.05)
ssGBLUP (Model 1)	17.89 (2.08)	41.59 (1.40)	-	0.30 (0.03)
ssGBLUP (Model 2)	9.93 (2.19)	56.44 (2.23)	3.38	0.19 (0.03)

### 3.3 SNPs and haplotypes associated with BCWD resistance

**FIGURE 3:**
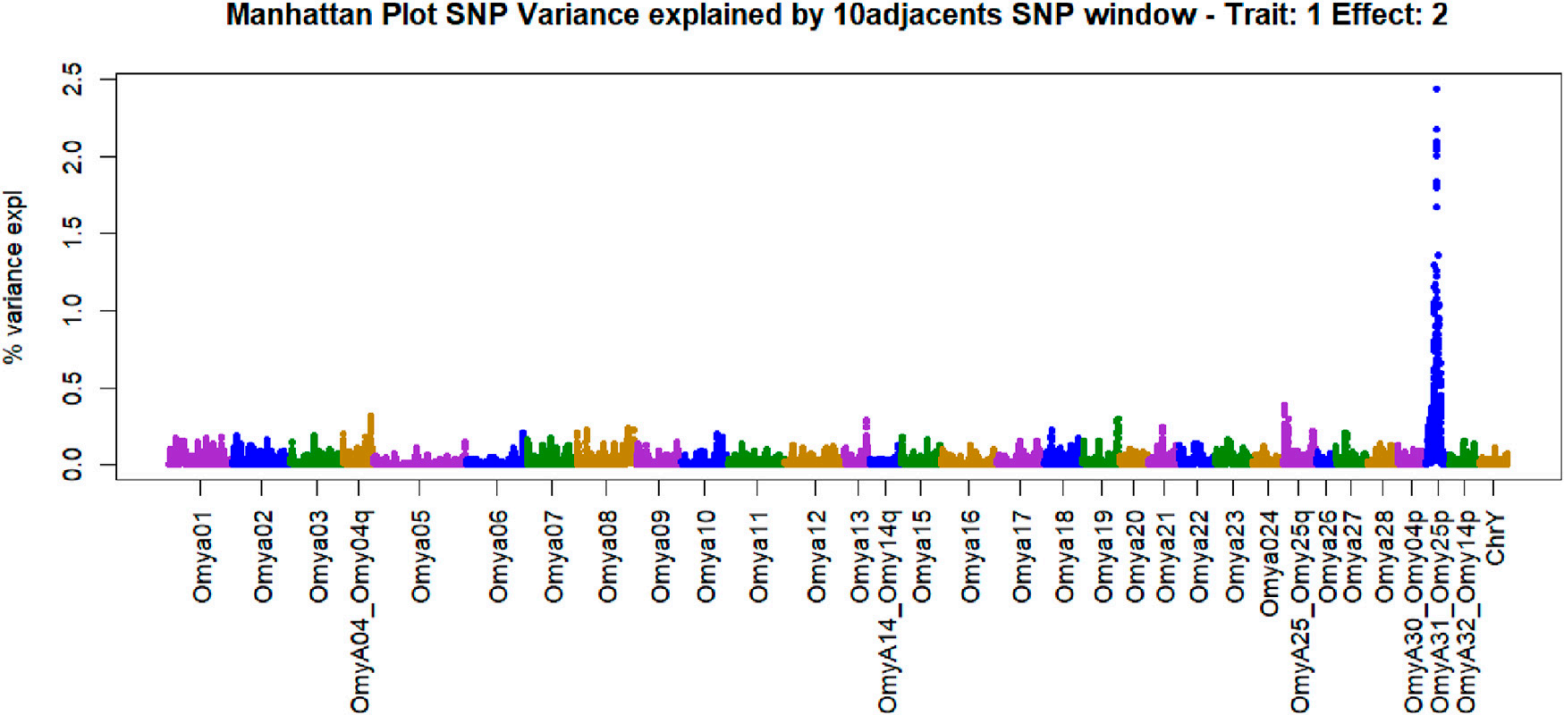
Manhattan plot of genome-wide association studies for resistance to bacterial cold-water disease in the 2020 year class of the February spawning commercial line of rainbow trout with ssGBLUP.

**FIGURE 4:**
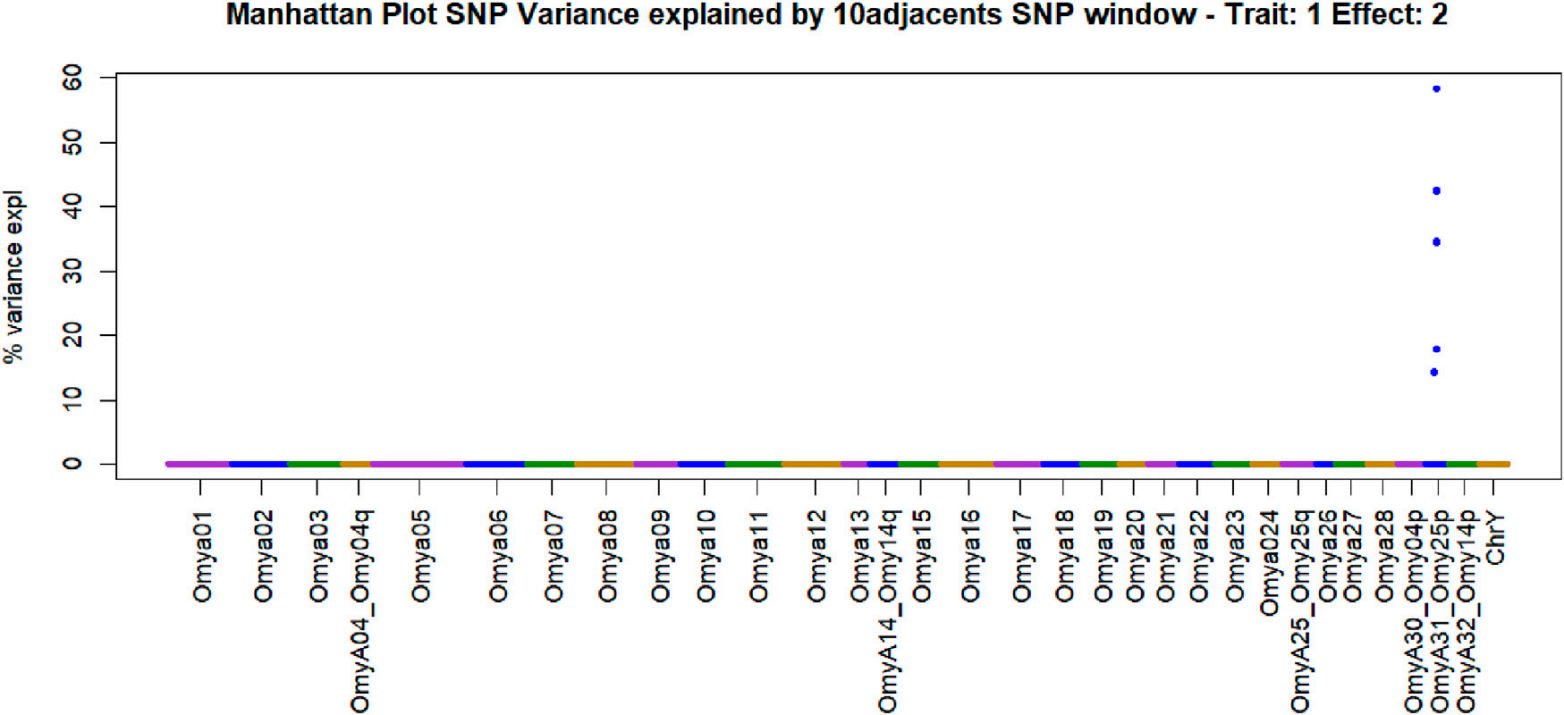
Manhattan plot of genome-wide association studies for resistance to bacterial cold-water disease in the 2020 year class of the February spawning commercial line of rainbow trout with weighted ssGBLUP under the NonlinearA weighting approach with a constant value of 2 and a limit of 50 from Iteration 10.

**FIGURE 5:**
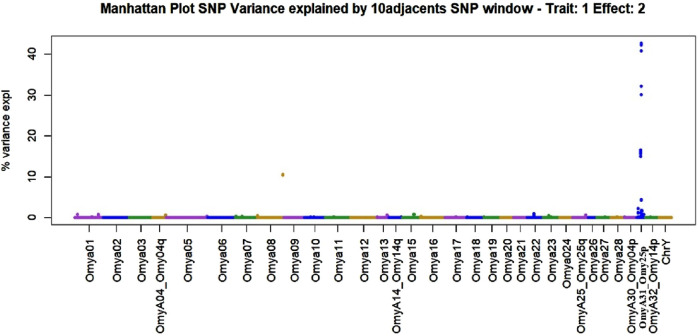
Manhattan plot of genome-wide association studies for resistance to bacterial cold-water disease in the 2020 year class of the February spawning commercial line of rainbow trout with weighted ssGBLUP under quadratic weights from Iteration 3. The model that generated this plot contained the fixed effect of haplotype (Model 2).

Manhattan plots containing GWAS outcomes from scenarios with complete data are reported in Figures 3–5. The quantitative trait loci associated with BCWD resistance are reported as the SNP window explaining more than 2% of the genetic variance (Tables 3, 4).

**TABLE 3:** Quantitative trait loci associated with bacterial cold-water disease resistance in the 2020 year class of the February spawning commercial line of rainbow trout using the complete dataset with NonlinearA weighting (constant = 2, limit = 50) for the 10th iteration with Model 1.

Omy	AGV(%)^a^	method	Physical map		Window flanking SNP		SNPs per window
			Start	End	Start	End	
OmyA04_Omy04q	2.05	Quadratic	43108924	46591609	Affx-88939405	Affx-88928160	10
OmyA08	4.28	Quadratic	82319447	82998371	Affx-88910955	Affx-88905101	10
OmyA31_Omy25p	3.25	Quadratic	23461749	23930336	Affx-88927562	Affx-88947015	10
OmyA31_Omy25p	14.34	Quadratic	25586167	25824203	Affx-88908807	Affx-88944742	10
OmyA31_Omy25p	3.80	Quadratic	25883294	26866871	Affx-88936445	Affx-88904970	10
OmyA31_Omy25p	14.41	NonlinearA	22690927	23508140	Affx-88905671	Affx-88927676	10
OmyA31_Omy25p	58.36	NonlinearA	25585611	25741004	Affx-88925154	Affx-88929795	10
OmyA31_Omy25p	18.02	NonlinearA	25883294	26866871	Affx-88936445	Affx-88904970	10

^a^ AGV (%)= percentage of total additive genetic variance explained by the window.

**TABLE 4:** Quantitative trait loci associated with bacterial cold-water disease resistance in the 2020 year class of the February spawning commercial line of rainbow trout using the complete dataset with quadratic weighting for the third iteration with Model 2 (haplotype model).

Omy	% Var^a^	Physical map		Window flanking SNP		SNPs per window
		Start	End	Start	End	
Omya08	10.67	82319447	82998371	Affx-88910955	Affx-88905101	10
OmyA31_Omy25p	2.26	11650678	12894284	Affx-88910335	Affx-88909023	10
OmyA31_Omy25p	16.56	21947534	22253737	Affx-88956678	Affx-88922824	10
OmyA31_Omy25p	42.76	22762507	23548257	Affx-88953068	Affx-88912866	10
OmyA31_Omy25p	4.47	23987971	30017598	Affx-88919974	Affx-88921090	10

^a^ % Var = percentage of total additive genetic variance explained by the window.

The SNPs removed from the “Total SNP” dataset to create the “Minus Common SNP” scenario were found in every chromosome except Omya16 and Omya24. In contrast, the SNPs removed to establish the “Minus Major SNP” scenario were specifically located in the OmyA31_Omy25p QTL region.

One haplotype was detected in the genomic regions highly associated with the trait. This haplotype had 19 SNPs, and its first SNP (Affx-88926696) was at position 25217123 bp on OmyA31_Omy25p chromosome, while the last SNP (Affx-88929795) was located on the same chromosome at position 25741004. The frequency of this “dominant haplotype” was 28.34%. Additionally, this haplotype was incorporated as a fixed effect in Model 2.

### 3.4 Accuracy and inflation for different validation approaches

**FIGURE 6:**
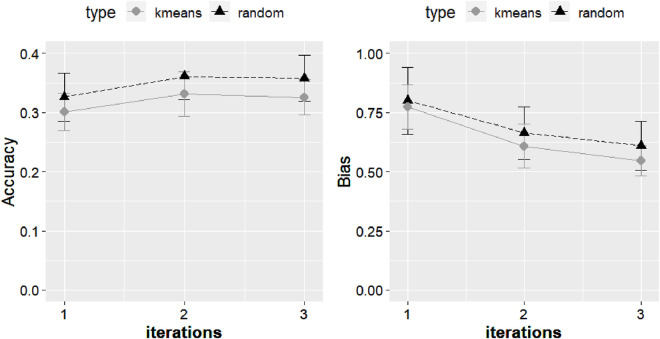
Accuracy expressed as the correlation between the (g)EBVs and corrected phenotypes and the regression coefficient of the corrected phenotype on the (g)EBVs (bias) for two 5-fold cross-validation scenarios (random and K-means) with Model 1 under weighted ssGBLUP performed for three iterations when using genomic information with the quadratic weighting approach. The values were averaged across the five replicates, and the error bars represent the standard deviations.

**FIGURE 7:**
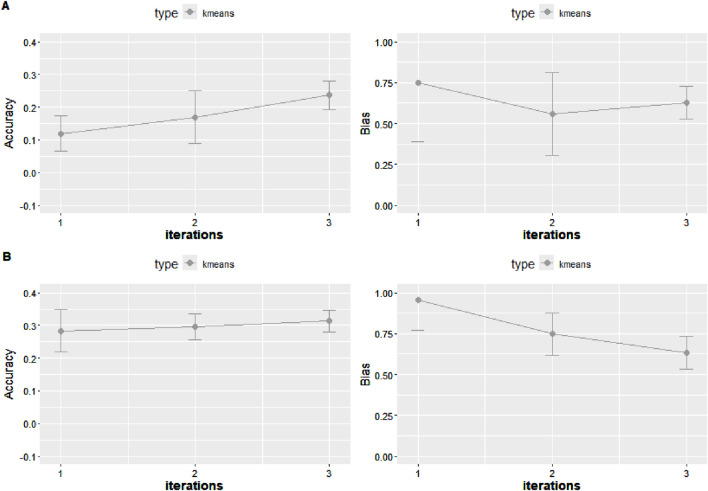
Accuracy expressed as the correlation between the (g)EBVs and corrected phenotypes and regression coefficient of the corrected phenotype on the (g)EBVs (bias) for two 5-fold cross-validation scenarios with K-means clustering under Model 2 under weighted ssGBLUP performed for three iterations when using genomic information with the quadratic weighting approach. The haplotype effect is added to (g)EBVs without the haplotype effect **(A)** and with the haplotype effect **(B)**. The values were averaged across the five replicates, and the error bars represent the standard deviations.

**FIGURE 8:**
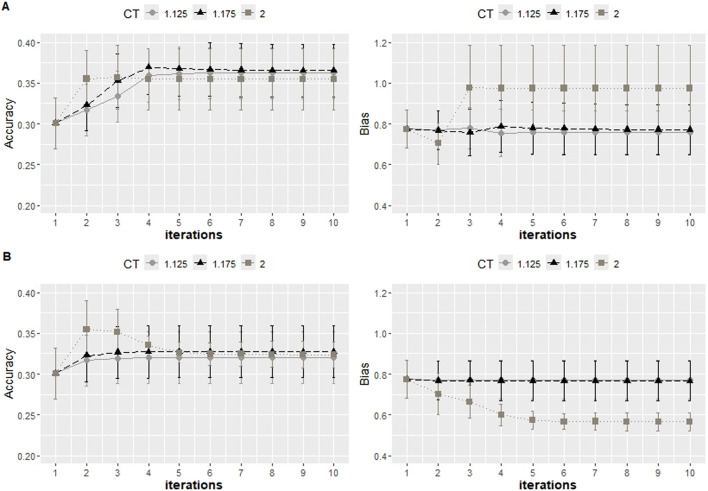
Accuracy expressed as the correlation between the (g)EBVs and corrected phenotypes and regression coefficient of the corrected phenotype on the (g)EBVs (bias) for two 5-fold cross-validation scenarios with K-means clustering under ssGBLUP under the nonlinear approach adopted with a constant value of 1.125, 1.15, and 2, with limits of 50 **(A)** and 10 **(B)** for ten iterations. The values were averaged across the five replicates, and the error bars represent the standard deviations.

**FIGURE 9:**
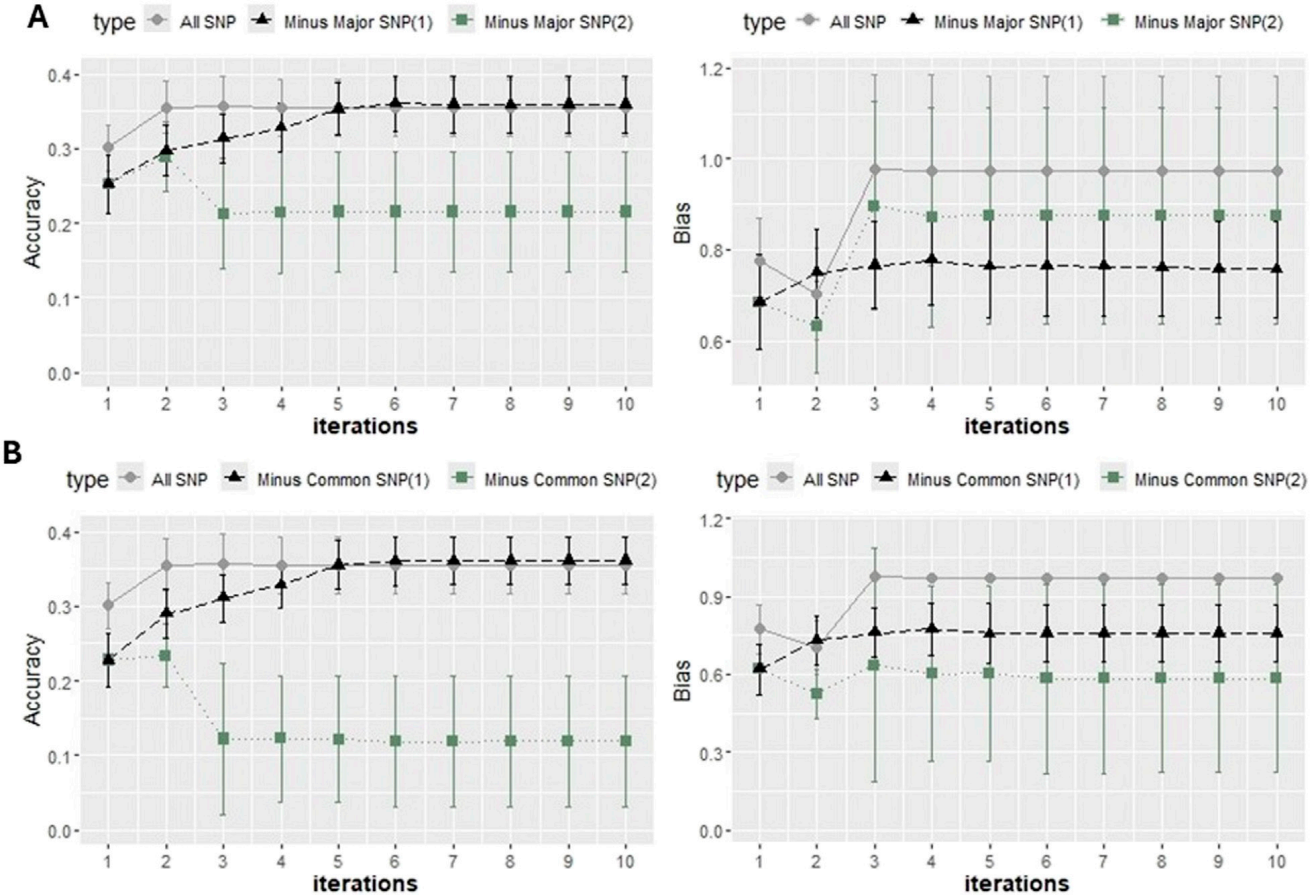
Accuracy expressed as the correlation between the (g)EBVs and corrected phenotypes and regression coefficient of the corrected phenotype on the (g)EBVs (bias) with two 5-fold cross-validation scenarios using K-means clustering under ssGBLUP with the NonlinearA approach adopted with a constant value of 1.125 and a limit of 10 **(A)** and a constant value of 2 and a limit of 50 **(B)**. All SNPs for the genotypic file included all markers after quality control. “Minus Major SNP” identifies that major SNPs were removed (−251 SNPs), and Minus Common SNP identifies that common SNPs were removed (−397 SNPs). The values were averaged across the five replicates, and the error bars represent the standard deviations.

The PBLUP approach with random cross validation yielded a prediction accuracy average (standard deviation) of 0.27 (0.03). With the K-means cross validation, the average PBLUP prediction accuracy was 0.26 (0.03). Prediction accuracy increased by 0.04 (15%) from PBLUP to ssGBLUP with the K-means validation and by 0.06 (22%) with random cross validation (Figure 6). The SNP weighting approaches increased accuracy gains up to a value of 0.36, which was 33% improvement over PBLUP with random cross validation and 38% over PBLUP with K-means cross validation (Figures 6–9).

The outcomes from the cross-validation scenarios using the quadratic weighting approach are illustrated in Figure 6, revealing a consistent pattern of lower accuracy in K-means validation than random cross validation. The accuracy increased from single-step GBLUP (ssGBLUP) to weighted single-step GBLUP (WssGBLUP) from 0.33 to 0.36 for the random cross validation with quadratic weighting, while for the K-means cross validation with quadratic weighting, accuracy improved from 0.30 to 0.33 (Figure 6). The constant 2 under the NonlinearA scenario caused accuracies to increase from 0.30 (ssGBLUP) to 0.35 (wssGBLUP) (Figure 8). Removing the haplotype from the G in Model 2 led to a substantial accuracy loss, which was partially recovered when weighting G (Figure 7). In the scenario where the haplotype effect was added to the GEBVs, the accuracy with ssGBLUP was 0.28, while the accuracy with wssGBLUP was 0.31.

The prediction accuracy of the haplotype effects alone was 0.23, which approaches the accuracy achieved with PBLUP. In this validation, one of the K-means groups presented a correlation of 0.83, due to a shift in haplotype frequency. This group was removed from the calculations in this scenario. When adding the haplotype effects to the PBLUP prediction, the accuracy increased to 0.3, which is similar to the results obtained with the entire SNP chip. The effect of carrying zero, one, or two copies of the haplotype was −1.34, −3.81, and +3.1 survival days, respectively. Surprisingly, the effect of this haplotype is not additive because having one copy resulted in lower survival than having no copies. This response may be due to the distribution of the other haplotypes in the QTL region, which, despite their lower frequency, do have varying effect levels on the survival phenotype.

Under PBLUP, the b1 for the random cross validation and K-means was 0.79 with both approaches and a standard deviation of 0.08 and 0.05, respectively. Additional values of b1 for the distinct approaches are reported in Figures 6–9. The GEBVs were inflated (b1 less than 1) for PBLUP (b1 = 0.79, sd = 0.09), and ssGBLUP with quadratic weighting further increased the inflation of GEBV (Figure 8). Including the haplotype effect in Model 2 resulted in less inflated GEBVs (b1 = 0.95) (Figure 7). NonlinearA with a constant of 2 and a limit of 50 significantly reduced inflation, converging on 0.97 when calculated with the K-means approach (Figure 8).

### 3.5 Accuracy and bias when excluding SNPs

When excluding “Major SNP” explaining more than 0.5% of the additive genetic variance from the genotypic file, the accuracy of prediction achieved with ssGBLUP decreased by 0.05 compared to when all markers are considered (Figure 7). In the same scenario, SNP weighting caused accuracy to decrease when using the constant and limit of 2 and 50, respectively. When more conservative parameters were used, the decrease in accuracy was less accentuated. To test whether the loss in accuracy was due to the reduction in panel density, one additional scenario in which 251 SNPs were randomly excluded was tested, and no impact on accuracy was observed (Results not shown).

After removing the “Common SNP,” there was a noticeable decrease in accuracy under NonlinearA with a constant of 2 and a limit of 50. The values from the last iteration ranged from 0.35 when all SNPs were included (Figure 8) to 0.12 after the elimination of the common SNPs (Figure 9) and to 0.21 after excluding major SNPs. When the approach was NonlinearA with a constant of 1.125 and a limit of 10, the initial decrease reported in Iteration 1 was compensated for when convergence was achieved.

## 4 Discussion

Previously, we identified two significant QTL for BCWD resistance on chromosomes Omy08 and Omy25p_OmyA31 in the Troutlodge May spawning line (Vallejo R. L. et al., 2017; 2022), which have been shown to be effective in a marker-assisted selection (MAS) breeding strategy in that line (Liu et al., 2018; 2022). For simplicity, we will refer to Omy25p_OmyA31 as OmyA31 for this discussion. In this study, we confirmed that the QTL located in the same genome regions on OmyA31 and Omy08 also exist in the genetically distinct February spawning line of Troutlodge, Inc. The OmyA31 QTL was found to have a larger effect on resistance to BCWD than the Omy08 QTL, and it has a dominant haplotype with possible implications for use in MAS in this commercially important rainbow trout line. Candidate genes co-located in this QTL genomic region were previously identified and implicated (Liu et al., 2022; Vallejo et al., 2022). Further analyses combining data from the two aquaculture populations are currently underway, aiming to further refine the QTL region in an attempt to pinpoint the causative gene and causative DNA sequence variant or variants.

Statistical and computational methods were applied to investigate the genetic basis of resistance to BCWD in this U.S. aquaculture line of rainbow trout. This study identified QTLs associated with BCWD survival while assessing outcomes from distinct models and approaches. Identifying QTL for BCWD resistance is expected to be more precise when SNP weighting approaches are used, due to the oligogenic nature of the trait in rainbow trout (Vallejo et al., 2018; 2022). The loss in accuracy when deleting major SNPs and dominant haplotype further validates the link between such markers and BCWD resistance. SNP weighting promoted greater accuracy estimates when all SNP markers were accounted for. Lastly, the results confirm that using different GWAS approaches might optimize QTL discovery and *in silico* validation.

### 4.1 Major genomic regions detected in GWAS

SNPs and QTL associated with BCWD resistance vary between populations (Vallejo R. L. et al., 2017). However, major QTL found in different analyses were validated for MAS (Liu et al., 2018). The implementation adopted in the current research computed the percentage of the additive genetic variance explained by windows of SNPs. However, the amount of variance to determine association varies across studies (Tiezzi and Maltecca, 2015; Han and Peñagaricano, 2016). In the GWAS with complete data in this study, we identified QTL as windows of 51 SNPs that explained more than 0.5% of the additive genetic variance. This approach aimed to identify windows harboring genomic regions associated with the trait (Marigorta and Navarro, 2013; Zhang et al., 2016). The two largest QTL peaks we detected in Omy25p (OmyA31) and in Omy08 agreed with previous reporting of QTL for resistance to BCWD in rainbow trout (Palti et al., 2015b; 2015a; Liu et al., 2018; Vallejo R. L. et al., 2017; 2022; Mathiessen et al., 2023).

An additional method to target genomic regions of importance for BCWD resistance was proposed in the current work. In this approach, subgroups were created using K-means clustering, and major SNPs present in all subgroups were identified. SNPs that explained more than 2% of the additive genetic variance and were consistent across subgroups were selected. Major SNPs were identified in all chromosomes with the exception of Omy16 and Omy24. QTL associated with resistance to BCWD were reported in previous studies on several chromosomes (Johnson et al., 2008; Wiens et al., 2013b; Campbell et al., 2014; Quillet et al., 2014; Vallejo et al., 2014; Vallejo R. L. et al., 2017; Liu et al., 2015; Palti et al., 2015b; Fraslin et al., 2019). Genes located in detected associated regions may provide insight into the biological mechanisms involved in BCWD resistance. A discussion of the potential candidate genes located near the large-effect QTL on chromosomes Omy8 and Omy25 can be found in previous publications (Liu et al., 2022; Vallejo et al., 2022). The approach used in this research used the percentage of variance explained to identify associations instead of p-values. A hypothesis testing using frequentist p-values in ssGBLUP was implemented by Aguilar et al. (2019). However, the use of p-values may not identify differences between regions regarding their effect. On the other hand, calculating the percentage of variance explained may underestimate some regions, especially when major QTLs exist and weighted approaches for the genomic relation matrix are used.

The sample selection used in this study might have biased the GWAS results and the estimates of the prediction accuracy of GBLUP models. Most of the samples used for GWAS and GBLUP estimates came from a single tank, where we used unbiased sampling of all the families from the population, but the samples that were genotyped from the other two tanks were pre-selected specifically from families with higher breeding values for other aquaculture production traits. The degree of potential bias due to pre-selected data is currently unknown but likely depends on the correlation of the other traits with resistance to BCWD and the intensity of selective breeding practiced.

Changes in the effects of SNPs and GWAS-associated regions may be a consequence of genetic diversity among populations, sample size variations, environmental influences on trait expression, the complexity of studied traits, different genotyping methods, population structures, statistical approaches, linkage disequilibrium variations, or inconsistencies in phenotype definitions (Uffelmann et al., 2021). Additionally, changes may also be a consequence of the small effective population size and subsequent small number of independent chromosome segments promoting collinearity and a high variance inflation factor for the estimators (Fragomeni et al., 2014; Dodd et al., 2022). Therefore, the gene associations discovered in single studies might not definitively represent the biology of the trait. Thus, there is a need for additional methods to validate SNP associations to strengthen the overall reliability and generalization of GWAS findings.

### 4.2 Variance components and genetic parameters

Differences were detected between the variance components obtained with the pedigree-based model (PBLUP) and genomic-enabled models (ssGBLUP). Lower heritability estimates were found with ssGBLUP. A similar pattern of reduced additive genetic variance and heritability with GBLUP models was noticed in previous studies (Vallejo R. et al., 2017; 2021). The ssGBLUP model considers additional information about the genetic structure of the population, which might allow for better estimating true genetic relationships, compensate for incomplete and inaccurate pedigree, and reduce bias (Aguilar et al., 2010; Cesarani et al., 2019; Misztal et al., 2020). The lower standard error in our heritability estimates generated with ssGBLUP may reflect a more stable and precise approach. Additionally, PBLUP assumes that the founder population is the base population and that the founders are unrelated (Falconer, 1960), while with marker-based models, the base population is the training population (Van Eenennaam et al., 2014). Nonetheless, PBLUP may pose issues for more closely related genotypes because the breeding value, which contributes to the phenotype, is more likely to be confounded with the Mendelian sampling term (Daetwyler et al., 2007). That occurs because gene copies inherited can still vary due to the randomness in genetic inheritance, and this artifact is expected to be less problematic when using genomic information for better estimating genetic relationships (Vallejo et al., 2021). Model 2, with the exclusion of the dominant haplotype from the genotype file under ssGBLUP, led to additional loss in the genetic variance component. This loss is an additional indicator of the large effect that this haplotype has on the BCWD resistance phenotypes.

### 4.3 Cross-validation scenarios

The K-fold validation process is largely adopted for assessment of genomic prediction approaches (Brøndum et al., 2011; Daetwyler et al., 2013; Schrauf et al., 2021). In this method, the genotyped individuals are divided into K random subsets, wherein each set is used for the training population of the other groups, and the omitted group is used for validation of the results. The K-means clustering method has been proposed to minimize the relationship between members of the training and validation sets that causes inflated accuracy (Saatchi et al., 2011). The advantage of using such an approach is that it would restrain the additional covariance that exists between variables due to kinship because K-means tends to cluster more homogeneous groups (Silva et al., 2016). Boddhireddy et al. (2014) observed that with K-means, cross-validation accuracies were consistently smaller than those of other approaches, similar to the findings of this study. Ventura et al. (2016) identified that clustering individuals based on genotypes could help find genotype errors and better reference populations. They also found that the number of animals in each cluster can impact the predictions and that groups with smaller sizes tend to have accuracy estimates with larger sampling variance. Additionally, the relationships between the training and the validation datasets can impact realized genomic selection accuracy (Saatchi et al., 2012). The random group cross validation increases the prediction accuracy by increasing the average relationship between the training and validation populations (Pszczola et al., 2012). Such values may result in overestimation of accuracy due to overfitting and consequently poor model selection.

### 4.4 Accuracy of weighted and unweighted ssGBLUP for different scenarios

Gains in prediction accuracy of BCWD resistance have been detected in SNP weighting and variable selection approaches with greater accuracy than unweighted models (Vallejo R. et al., 2017). Such differences may be attributed mainly to the oligogenic genetic architecture of the trait in study. The same pattern is observed in the results of this work. Santana et al. (2023) observed a decline in accuracy for traits controlled by 1000 QTL and 2000 QTL when using quadratic weights, while a trait controlled by 100 QTL experienced accuracy gains with the same method. The adequate weighting strategy varies based on trait genetic architecture (Lopes et al., 2017; Lourenco et al., 2017). NonlinearA weights have been shown to be more stable than quadratic weights (Zhang et al., 2016; Fragomeni et al., 2019). However, NonlinearA weighting depends on predetermined equation parameters (VanRaden, 2008). Incorrect parameters lead to low accuracy and biased predictions (Santana et al., 2023).

The strategy to remove major SNPs in this study was adopted to enhance the understanding of the association of such SNPs with BCWD resistance. How accuracy was affected with the different SNP arrays was compared, and the cases where major SNPs and the dominant haplotype were removed caused accuracy to decrease. Weighting SNPs in the “Minus Major SNP” scenario further decreased accuracy, indicating that the markers removed are likely of higher influence, especially when weighting approaches allowed a large departure from normality. When removing major SNPs, the response due to weighting is expected to be close to the response of a polygenic trait. For this reason, quadratic weights and extreme values in the NonlinearA equation resulted in low accuracy. In a similar approach, Vallejo et al. (2018) detected a sharp decline in the accuracy of the BayesB predictions between using the higher density panel without Omy8 and Omy25 and using a 500 SNP panel without Omy8 and Omy25. In our study, the random removal of a few hundred SNPs from the array with 36,000 did not impact the prediction accuracy (results not shown), demonstrating that the loss in accuracy is not due to reduced density of the panel. Similarly, Kriaridou et al. (2020) observed that, at levels above 2,000 SNPs, changes in panel densities did not significantly affect the accuracy of predictions across various aquaculture species. The research found consistent prediction accuracies across a range of panel densities, spanning from 2,000 to 7,000, with increments of 100 SNPs per panel.

Because of the complexity of traits’ genetic architecture, many variable selection approaches have been implemented and demonstrated relevant findings even for polygenic traits. MacLeod et al. (2016) investigated the use of BayesR and Bayes RC while exploiting biological priors and inclusion of putative variants with distinct distributions for the SNP effects assumed. BayesR employs a Markov chain Monte Carlo methodology to estimate variant effects using prior biological knowledge. These effects are modeled as a mixture distribution comprising four normal distributions, with one of these representing a null distribution. BayesRC shared a similar approach with BayesR except that *a priori* independent biological information was used to allocate each variant to a specific class. BayesRC improved the accuracy of QTL discovery and genomic prediction and was shown to be useful while accounting for biological knowledge regarding functional regions of the genome. Such approaches could be beneficial for traits with a polygenic architecture but with a few major regions, such as BCWD resistance.

The gains in accuracy in PBLUP obtained by adding the haplotype effect were higher than those described by Lopes et al. (2017). Such differences are likely due to the magnitude of the QTL identified in this study. However, the population structure may also have an influence on those findings, and more research on the causality of the QTL should be performed before commercial implementation of a marker-assisted selection program. The current results encourage the inclusion of the single marker in a pedigree-based breeding program. However, a selection program based on a single genomic region may lead to a quick fixation of the allele and haplotype within populations, so constant genetic screening is very important if the MAS approach is being considered.

Removing major SNPs caused subsequent losses in accuracy and gave additional insight into the association between genetic markers and the studied trait. The strategy of selecting consistent major SNPs across more homogeneous groups served to bolster confidence in the significance of specific genetic markers. Identifying similar associations across diverse genetic groups such as breeds, lines, and families serves to further enhance the credibility of variant causality (van den Berg et al., 2016). Hence, clustering genotypes and identifying more homogeneous groups for cross validation may help in the process of establishing associations. Removing a major QTL and including it as a fixed effect caused the accuracy of prediction to decrease more than in the other scenarios, indicating an important association between the dominant haplotype and BCWD resistance. However, that accuracy can be recovered when the haplotype effect is included in the breeding value. Therefore, incorporating QTL information into genomic selection methods is a promising application. Previously, the QTL in chromosome Omy25 was shown to be potentially useful for marker-assisted selection in other commercial rainbow trout lines, resulting in accuracies higher than family-based selection (Liu et al., 2018; 2022; Vallejo et al., 2022; Mathiessen et al., 2023). MAS stands out as a viable and cost-effective alternative for selective breeding to improve resistance to BCWD in rainbow trout because of the cost savings in genotyping and because it does not require recurring phenotyping for model retraining in each generation.

## 5 Conclusion

In this study, a successful implementation of GWAS and genomic prediction for resistance to BCWD was achieved in an important commercial rainbow trout line that has not been previously investigated for this trait, resulting in higher prediction accuracy through genomic selection. Comparative analysis of different cross-validation methods suggested that K-means reduced overfitting. The incorporation of SNP weighting further substantiated the underlying oligogenic architecture of the trait under investigation. Bias was minimal in ssGBLUP when the major effect haplotype on Omy25/OmyA31 was included as a fixed effect and in weighted ssGBLUP with optimized equation parameters. Additionally, a decrease in accuracy upon the exclusion of major SNPs was observed and serves as supplementary evidence of their effect on phenotypes. Alternative validation methodology unveiled a new set of major-effect SNPs. Removing a major haplotype from the SNP panel resulted in a substantial reduction in the prediction accuracy and modification of the genetic architecture. Conversely, the inclusion of the major haplotype effect as a fixed effect illustrated its potential utility in marker-assisted selection as previously reported for two other commercial lines of rainbow trout.

## Data Availability

The original contributions presented in the study are included in the article/supplementary material; further inquiries can be directed to the corresponding author.
